# A greedy alignment-free distance estimator for phylogenetic inference

**DOI:** 10.1186/s12859-017-1658-0

**Published:** 2017-06-07

**Authors:** Sharma V. Thankachan, Sriram P. Chockalingam, Yongchao Liu, Ambujam Krishnan, Srinivas Aluru

**Affiliations:** 1 Department of Computer Science, University of Central Florida, Orlando, 32816 FL USA; 2 Institute for Data Engineering and Science, Georgia Institute of Technology, Atlanta, 30332 GA USA; 3 School of Computational Science and Engineering, Georgia Institute of Technology, Atlanta, 30332 GA USA; 4 School of Electrical Engineering and Computer Science, Louisiana State University, Baton Rouge, 70703 LA USA

**Keywords:** Alignment-free methods, Sequence comparison, Phylogeny reconstruction

## Abstract

**Background:**

Alignment-free sequence comparison approaches have been garnering increasing interest in various data- and compute-intensive applications such as phylogenetic inference for large-scale sequences. While *k*-mer based methods are predominantly used in real applications, the average common substring (ACS) approach is emerging as one of the prominent alignment-free approaches. This ACS approach has been further generalized by some recent work, either greedily or exactly, by allowing a bounded number of mismatches in the common substrings.

**Results:**

We present ALFRED-G, a greedy alignment-free distance estimator for phylogenetic tree reconstruction based on the concept of the generalized ACS approach. In this algorithm, we have investigated a new heuristic to efficiently compute the lengths of common strings with mismatches allowed, and have further applied this heuristic to phylogeny reconstruction. Performance evaluation using real sequence datasets shows that our heuristic is able to reconstruct comparable, or even more accurate, phylogenetic tree topologies than the kmacs heuristic algorithm at highly competitive speed.

**Conclusions:**

ALFRED-G is an alignment-free heuristic for evolutionary distance estimation between two biological sequences. This algorithm is implemented in C++ and has been incorporated into our open-source ALFRED software package (http://alurulab.cc.gatech.edu/phylo).

## Background

Accurate estimation of the evolutionary distance between two sequences is fundamental and critical to phylogenetic analysis aiming to reconstruct the correct evolutionary history and estimate the time of divergence between species. One popular approach to evolutionary distance estimation relies on sequence alignment. Typically, the pipeline for alignment-based phylogenetic inference generally works by three steps. Firstly, we perform all-to-all pairwise sequence alignment to gain a pairwise distance matrix for the input sequences. The evolutionary distance between two sequences in the matrix is typically inferred from an optimal alignment, e.g. equal to one minus percent identity in the optimal alignment. Secondly, we construct a guide tree from the pairwise distance matrix and then conduct progressive alignment of multiple sequences following the order determined by the guide tree. Finally, we infer a phylogenetic tree from the resulting multiple alignments using a tree inference program which can be distance-, parsimony-, bayesian, or likelihood-based. Nevertheless, it needs to be stressed that we could also choose to construct a phylogenetic tree directly from the pairwise distance matrix computed in the first step, using some distance-based tree construction algorithm such as unweighted pair group method with arithmetic mean (UPGMA) [1] or neighbor-joining (NJ) [2].

Although they may have high accuracy, alignment-based approaches involve high computational cost, thus resulting in slow speed. This is because pairwise alignment using dynamic programming has a quadratic complexity with respect to sequence length. This is even more challenging when constructing the phylogenetic tree for a large number of sequences, especially long sequences (e.g. eukaryotic genomes). In this case, some research efforts have been devoted to accelerating the tree construction using high performance computing architectures [3–6]. In addition to acceleration, as an alternative to alignment-based approaches, alignment-free approaches emerge and become popular, mainly owing to their speed superiority. For instance, given a collection of *d* sequences of average length *n*, the time complexity for pairwise distance matrix computation can be as high as *O*(*d* ^2^ *n* ^2^) when using pairwise alignment. In contrast, by using alignment-free exact *k*-mer (a *k*-mer is a string of *k* characters) counting, the whole computation can be done in *O*(*d* ^2^ *n*) time, significantly reducing the run-time by a factor of *n*. Moreover, alignment-free approaches are capable of overcoming some difficulties, which challenge alignment-based approaches, such as genetic recombination and shuffling during the evolution process.

A variety of alignment-free approaches have been proposed, most of which are based on the concept of sequence seeding that extracts fixed- or variable- length substrings from a given sequence. Based on fixed-length seeding, there are two kinds of alignment-free approaches: exact *k*-mer counting [7] and spaced *k*-mer counting [8]. For the exact *k*-mer counting approach, it builds a *k*-mer frequency (or occurrence) vector for each sequence and computes the pairwise distance using some distance measure based on the frequency vectors. Example distance measures include Euclidean distance [9], Kullback-Lebler divergence [10] and the one proposed by Edgar [11]. The Edgar’s distance measure models the similarity between two sequences as the fraction of exact *k*-mers shared by them, and then computes the pairwise distance by subtracting the similarity value from one. This distance measure has been shown to be highly related to genetic distance and has been used in other applications like metagenomic sequence classification [12]. For the spaced *k*-mer counting approach, it allows character mismatches between *k*-mers at some predefined positions and usually employs multiple pattern templates in order to improve accuracy.

Based on variable-length seeding, there are three kinds of approaches: the average common substring (ACS) method [13], the *k*-mismatch ACS (k-ACS) method [14, 15] and the mutation distances (*K*_*r*_) [16]. The distance based on these methods can be computed using suffix trees/arrays. Given two sequences, the ACS method first calculates the length of the longest substring that starts at each position *i* in one sequence and matches some substring of the other sequence. Subsequently, it averages and normalizes all of the lengths computed to represent the similarity of the two sequences. Finally, the resulting similarity value is used to compute the pairwise distance. The time complexity of the ACS method is directly proportional to the sum of lengths of the two sequences.

In contrast, the k-ACS method computes the pairwise distance by finding substring pairs with upto *k* mismatches, given two sequences. Specifically, instead of determining the longest common substrings, the k-ACS method aims to find the longest substring starting at each position in one sequence and matching some substring in the other sequence with upto *k* mismatches. The mutation distances is closely related to ACS, where the difference is only in the conversion from the similarity value to a pairwise distance.

Unlike the ACS method, the solutions to the k-ACS method involves high computational cost. For example, an algorithm given by Leimeister and Morgenstern [14] takes *O*(*k* *n* ^2^) time in the worst case, which is certainty not a suitable replacement of alignment based methods. However, they proposed a faster algorithm, namely kmacs, that computes an approximation to k-ACS based distance. Another algorithm by Apostolico et al. runs in *O*(*n* ^2^/ log*n*) time [17]. This raises an open question, whether the exact k-ACS based distance can be computed in strictly sub-quadratic time. Initial attempts were focused on the special case of *k*=1 [18, 19]. Later, Aluru et al. [15, 20] positively answered this question by presenting an algorithm with a worst case run time of *O*(*n* log*k* *n*) for any constant *k*. The algorithm is much more complicated than the original ACS method and even the k-ACS approximation by [14]. Moreover the practical variant of this algorithm can get quite slow for even moderately large values of *k* due to its exponential dependency on *k* [21]. However, this algorithm has its merit as the first sub-quadratic time algorithm for exact k-ACS computation for any positive integer *k*. A recently proposed algorithm by Pizzi is based on filtering approaches [22]. In summary, on one hand, we have a fast approximation algorithm [14] and on the other hand, we have an exact (theoretical) algorithm [15], that might work well for small values of *k* in practice. Inspired by both algorithms, we introduce a new **g**reedy heuristic for **al**ignment-**fre**e **d**istance estimation, named ALFRED-G. The heuristic is implemented in C++ and has been incorporated into our open-source ALFRED software package (http://alurulab.cc.gatech.edu/phylo).

We use X and Y to denote the two sequences to be compared. The length of sequence X is denoted by |X|, its *i*th character by X[*i*], and the substring that starts at position *i* and ends at position *j* by X[*i*…*j*]. For brevity, we use X _*i*_ to denote the suffix of X starting at *i*. The total length of X and Y is denoted by *n*. A key data structure in our algorithm is the generalized suffix tree (*GST*). The *GST* of X and Y is a compact trie of all suffixes of X and Y. It consists of *n* leaves and at most *n*−1 internal nodes. Corresponding to each leaf, there is a unique suffix of X or Y. The edges are labeled with a sequence of characters. The *string-depth* of a node *u* is the length of the string obtained by concatenating the edge labels on the path from the root of *GST* to *u*. The space and the construction time of *GST* are *O*(*n*) [23]. For any (*i*,*j*) pair, |*LCP*(X _*i*_,Y _*j*_)|, the length of the longest common prefix of X _*i*_ and Y _*j*_ is same as the string-depth of the lowest common ancestor node of the leaves corresponding to X _*i*_ and Y _*j*_. Using *GST*, we can compute it in constant time. Also, we can compute |*LCP*_*k*_(X _*i*_,Y _*j*_)|, the length of the longest common prefix of X _*i*_ and Y _*j*_ with first *k* mismatches ignored, in *O*(*k*) time as follows. Let *z*=|*LCP*(X _*i*_,Y _*j*_)|, then for any *k*≥1,

1 $$ \left|\mathsf{LCP}_{k}\left(\mathsf{X}_{i}, \mathsf{Y}_{j}\right)\right| = z+1+\left|\mathsf{LCP}_{k-1}\left(\mathsf{X}_{i+z+1}, \mathsf{Y}_{j+z+1}\right)\right|  $$

### Problem definition

The *k*-mismatch average common substring of X w.r.t. Y, denoted by *ACS*_*k*_(X,Y) is defined as the average of the length of the prefix of a suffix of X, that appears as a substring of Y within hamming distance *k*. Specifically, let *λ*_*k*_(*i*)= max*j*|*LCP*_*k*_(X _*i*_,Y _*j*_)|, then

2 $$ \mathsf{ACS}_{k}(\mathsf{X}, \mathsf{Y}) = \frac{1}{|\mathsf{X}|}\sum_{i=1}^{|\mathsf{X}|}\lambda_{k}(i)  $$

The distance *Dist*_*k*_(X,Y), based on *ACS*_*k*_ is given below [13, 14].

3 $${} \begin{array}{ll} \mathsf{Dist}_{k}(\mathsf{X},\mathsf{Y}) &= \frac{1}{2} \left(\frac{\log |\mathsf{Y}|} {\mathsf{ACS}_{k}(\mathsf{X},\mathsf{Y})}+\frac{\log |\mathsf{X}|}{\mathsf{ACS}_{k}(\mathsf{Y},\mathsf{X})}\right) -\left(\frac{\log |\mathsf{X}|}{|\mathsf{X}|}+\frac{\log |\mathsf{Y}|}{|\mathsf{Y}|}\right)\\ \end{array}  $$

## Methods

### Approximating *ACS*_*k*_(·,·)

It is observed that *ACS*_*k*_(·,·) can be easily computed in *O*(*n* ^2^ *k*) time via |X|×|Y| number of |*LCP*_*k*_(·,·)| queries, which is clearly not affordable. The first attempt to circumvent this issue was made by Leimeister and Morgenstern [14], who presented a heuristic method, named kmacs, that quickly computes an approximation to *ACS*_*k*_(X,Y). The key idea is to replace *λ*_*k*_(*i*) with *λ* *k*′(*i*) in the equation for *ACS*_*k*_, where *α*_*i*_=*a* *r* *g* max*j*|*LCP*(X _*i*_,Y _*j*_)| and $\lambda _{k}'(i) =|\mathsf {LCP}_{k}(\mathsf {X}_{i},\mathsf {Y}_{\alpha _{i}})|\phantom {\dot {i}\!}$. Using *GST*, we can compute *α*_*i*_ for all values of *i* in *O*(*n*) time. Therefore, *λ* *k*′(*i*) for all values of *i* and the corresponding distance can be easily obtained in *O*(*n* *k*) time. Note that the ratio of *λ*_*k*_(*i*) to *λ* *k*′(*i*) can be as high as *Θ*(*n*). Nonetheless, it has been shown that for most practical cases, the average of the latter can serve as a good approximation to the average of the former.

### Our approach

The idea is to follow a simple adaptation of Aluru et al.’s exact algorithm [15] for 1-mismatch case and then use the heuristic approach by Leimeister and Morgenstern [14] to extend the result to *k*-mismatch. Specifically, our approximation to *ACS*_*k*_ is obtained by replacing *λ*_*k*_(*i*) in the equation for *ACS*_*k*_ by *λ* *k*″(*i*), where *β*_*i*_=*a* *r* *g* max*j*|*LCP*_1_(X _*i*_,Y _*j*_)| and $\lambda _{k}''(i) =|\mathsf {LCP}_{k}(\mathsf {X}_{i},\mathsf {Y}_{\beta _{i}})|\phantom {\dot {i}\!}$. To compute *β*_*i*_ for *i*=1,2,…,|X|, we first construct *GST* and an array *A*[1,|X|]. Then for each internal node *u* in *GST*, process the set $\mathcal {S}(u)$ of suffixes corresponding to the leaves in the subtree of *u*. Let *h* be the *string-depth* of *u*. Then (*h*+1) is the first position, in which the prefixes of two suffixes in $\mathcal {S}(u)$ can differ. We sort all suffixes in $\mathcal {S}(u)$ by treating the (*h*+1)th character all suffixes to be identical, or equivalently first (*h*+1) characters to be the same. To do so, we follow the steps below:

- Map each $\mathsf {X}_{i} \in \mathcal {S}(u)$ to a pair (X _*i*_,*k* *e* *y*), where *key* is the lexicographic rank of the suffix X _*i*+*h*+1_ among all suffixes of X and Y. In other words, *key* is the lexicographic rank of the suffix obtained by deleting the first (*h*+1) characters of X _*i*_. Using *GST*, we can compute *key* in constant time.
- Likewise, map each $\mathsf {Y}_{j} \in \mathcal {S}(u)$ to a pair (Y _*j*_,*k* *e* *y*), where *key* is the lexicographic rank of Y _*j*+*h*+1_ among all suffixes of X and Y.
- Sort all pairs in the ascending order of *key*.
- For each pair (X _*i*_,·), find the closest pairs, say (Y _*a*_,·) and (Y _*b*_,·), towards the left and right side (if they exist) that are created from a suffix of Y, and update *A*[*i*]←*a* *r* *g* max*j*∈{*a*,*b*,*A*[*i*]}|*LCP*_1_(X _*i*_,Y _*j*_)|.

After processing all internal nodes as described above, compute the following and report it as our approximation to *ACS*_*k*_(X,Y)

$$\frac{1}{|\mathsf{X}|} \sum_{i=1}^{|\mathsf{X}|} \lambda^{\prime\prime}_{k}(i) = \frac{1}{|\mathsf{X}|} \sum_{i=1}^{|\mathsf{X}|} \left|\mathsf{LCP}_{k}\left(\mathsf{X}_{i},\mathsf{Y}_{\beta_{i}}\right)\right| $$

It can be easily verified that *A*[*i*] will be correctly updated to *β*_*i*_ while processing the lowest common ancestor node of the leaves corresponding to X _*i*_ and $\mathsf {Y}_{\beta _{i}}$. The overall run time is $nk+\sum _{u} |\mathcal {S}(u)|\log |\mathcal {S}(u)|=O(nk+nH\log n)$, where *H* is the height of *GST* and its expected value is *O*(log*n*) [24].

### Implementation

ALFRED-G is implemented in C++ and is incorporated in our open-source ALFRED software package (http://alurulab.cc.gatech.edu/phylo). This algorithm takes a collection of sequences as input and computes an approximation to *ACS*_*k*_(·,·) for all pairs of sequences. For this, we have used the open-source libdivsufsort library [25] to construct the suffix array (SA) and have used the implementations in the SDSL library [26] to build the corresponding LCP table (using the Kasai algorithm [27]) and the range minimum query (RMQ) table (using the Bender-Farach’s algorithm [28]). (Note that the operations on a suffix tree can be simulated using the corresponding SA, inverse SA, LCP array and RMQ table). The SDSL library has support for using bit compression techniques to reduce the size of the tables and arrays in exchange for slower query time. However, we don’t compress these data structures, and instead we have used 32-bit integers for indices as well as prefix lengths.

## Results and discussion

### Benchmark datasets

We have assessed the performance of ALFRED-G for the reconstruction of phylogenetic trees by using three sequence datasets, which contain prokaryotic DNA sequences, eukaryotic DNA sequences, and protein sequences, respectively. The prokaryotic sequence dataset consists of 27 Primate mitochondrial genomes, which was previously studied by [16] in order to assess the performance of alignment-free approaches for phylogenetic tree reconstruction. In the study, a reference tree was constructed based on a multiple alignment of the sequences.

The eukaryotic sequence dataset is constructed by Newton et al. [29] from 32 Roseobacter genomes, by extracting 70 universal single-copy genes for the 32 genomes with each gene being completely sequenced in all genomes and having no ambiguous start/stop sites. The 70 genes for each genome are, subsequently, concatenated and aligned with ClustalW in Geneious 4.0 (available from http://www.geneious.com) using *Escherichia coli* K12 substrain MG1655 as the outgroup. The multiple sequence alignment file is available at http://alurulab.cc.gatech.edu/phylo, from which the raw sequences corresponding to the 32 Roseobacter genomes are extracted and then used in our study. In our study, we have used the phylogenetic tree presented in Newton et al. [29] as the reference tree.

The protein sequence dataset is taken from BAliBASE (v3.0) [30], which is popular benchmark dataset for multiple sequence alignment. We have used 218 sets of protein sequences in BAliBASE, and constructed the reference trees from the corresponding reference alignments using the *proml* program available in PHYLIP [31], which implements the Maxmimum Likelihood method. For each of the parameter selected for our experiments, we report the average RF-distance of the 218 trees constructed from this set.

### Phylogenetic tree construction and comparison

Given a set of *d* sequences, we first compute the distance between any sequence pair and then construct a pairwise distance matrix of size *d*×*d*. Subsequently, the neighbor-joining (NJ) algorithm [2] is applied on the pairwise distance matrix to reconstruct the phylogenetic tree, where the neighbor program in PHYLIP is used. Finally, the topology of the tree is compared with the reference tree using the Robinson-Foulds (RF) distance metric, where the treedist program in PHYLIP is used to compute the RF distance between two trees. Note that the lower the RF distance is, the better the tree topology matches. In particular, if the RF distance equals zero, it means exact topology match between the two trees.

All experiments are preformed in an Apple Macbook Pro (Mid-2012 model) running Mac OS 10.10.4 (OS X Yosemite). The machine features a 2.9 GHz dual-core Intel Core i7-3667U processor with 4MB L3 cache and 8GB RAM.

### Performance comparison

As our method is closely related to kmacs, we compared the performance of ALFRED-G with kmacs in terms of speed and accuracy (based on RF-distance) for different values of *k*, ranging from 0 to 9. Note that for *k*=0, both kmacs and ALFRED-G are the same as the ACS method.

**Fig. 1:**
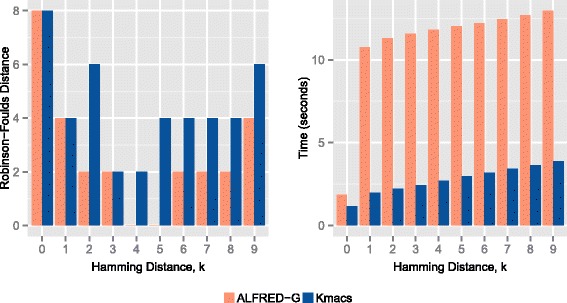
RF distance and run-time plots for the prokaryotic dataset

**Fig. 2:**
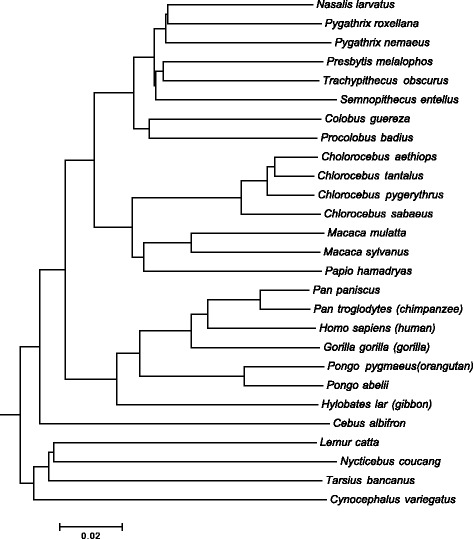
Tree generated by ALFRED-G for the prokaryotic dataset with *k*=4

Figure 1 shows the results for the prokaryotic dataset. It can be observed that for all values of *k*, ALFRED-G provides either the same or better accuracy (in terms of RF distance). Interestingly, for *k*=4 and 5, the phylogenetic tree created based on ALFRED-G coincides exactly with the reference tree (see Fig. 2). We notice that the only other alignment-free method, that was able to recreate this exact reference tree is the recently proposed spaced-seed method [8] (but needs careful parameter turning).

**Fig. 3:**
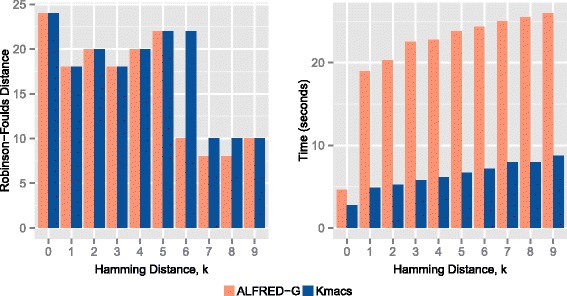
RF distance and run-time plots for the eukaryotic dataset

**Fig. 4:**
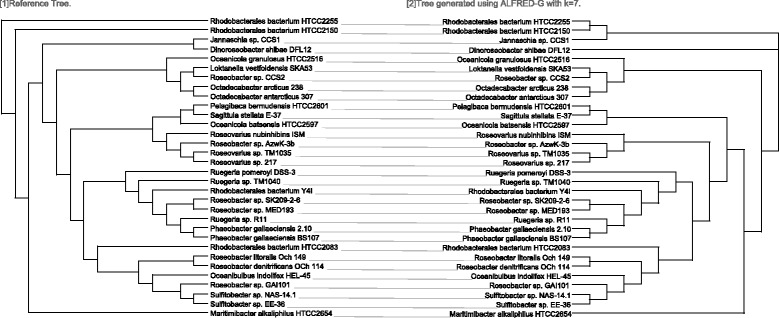
Reference tree and the tree generated by ALFRED-G for the eukaryotic dataset with *k*=7 (RF distance = 8)

Figure 3 shows the results for the eukaryotic dataset. Likewise, our RF distance is never worse than that obtained by kmacs. In particular, when setting *k*=6,7 and 8, our RF distance is lower, indicating better performance. Figure 4 shows the topological comparison between the tree generated by our approach and the reference tree, which is generated by the Dendroscope software [32].

**Fig. 5:**
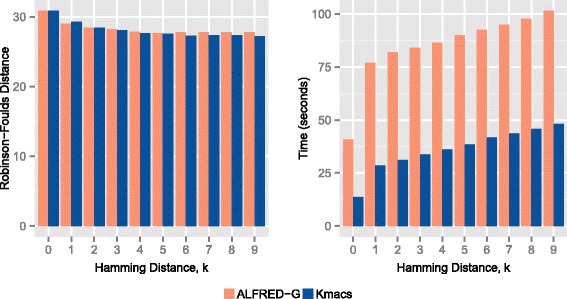
RF distance and run-time plots for the BAliBASE protein dataset

Figure 5 shows the results for the protein dataset. Here both ALFRED-G and kmacs gave almost the same RF score for each value of *k*. As expected, ALFRED-G is slower than kmacs (by a factor of 2 to 4), however the difference in run-time is independent of *k*.

In the earlier work by Leimeister and Morgenstern [14], it has been show that kmacs and spaced-seed [8] are superior to other alignment-free methods, when applied to the aforementioned three datasets. Our experiments show that ALFRED-G is comparable and often more accurate than kmacs, albeit involving higher computational cost. It needs to be mentioned that the comparison with spaced-seed is not as straightforward as with kmacs, because spaced-seed has different input parameters and requires tedious pattern templates tuning. Nevertheless, we have carefully evaluated spaced-seed based on the suggestions from [8]. Our evaluation shows that spaced-seed is able to recover the entire reference tree (i.e. RF distance = 0) for the prokaryotic dataset, in just 4 seconds. However, for the rest, the performance of spaced-seed is roughly comparable to both ours and kmacs.

## Conclusions

In this paper, we have introduced a greedy alignment-free approach to estimating the evolutionary distance between two sequences. The core of the heuristic is to identify a 1-mismatch longest substring in sequence Y that appears as a prefix of any given suffix in sequence X, and vice versa. This heuristic has been further applied to reconstruct the phylogenetic tree, given a collection of sequences that are believed to be close enough and have some evolutionary relationship between them. The performance of our heuristic has been evaluated using three real datasets: one prokaryotic dataset, one eukaryotic dataset and one protein dataset, in terms of tree-topology RF score and speed. Our experimental results show that our heuristic can exactly reconstruct the same phylogenetic tree topology with the reference tree for the prokaryotic dataset, whereas kmacs cannot. On the remaining two datasets, our heuristic also demonstrates comparable or even better performance than kmacs. As for speed, our heuristic is slightly slower than kmacs.

Although our heuristic has been shown effective for phylogenetic inference, there are still some limitations that could be improved in the future. Firstly, our heuristic assumes an evolution model having only mismatches, not involving insertions or deletions, for simplicity. This model may not exactly fit the real evolutionary process given a collection of sequences. Nevertheless, our performance evaluation has shown that even though there are some insertions or deletions between sequences (observed from multiple sequence alignment), their evolutionary distances can still be estimated with reasonable accuracy using our heuristic. However, it should be noted that the existence of insertions or deletions may cause our heuristic to underestimate the similarity values, i.e. *ACS*_*k*_(·,·), between sequences, thus overestimating their distances, i.e. *Dist*_*k*_(X,Y).

Secondly, our heuristic assumes that the homologous regions between two sequences are on the same strand. Actually, this is not always the case. Given a homologous region, the substring in sequence X may have an opposite strand to the corresponding homology in sequence Y. In this case, directly applying our heuristic to such sequences may overestimate the distance, since these homologies with opposite strands are not counted in the computation of similarity values. In some sense, we would expect that the estimation accuracy of alignment-free approaches could be further improved by incorporating support for strand differences in homologies.

Thirdly, our heuristic has only used Eq. (3) to estimate the distance from the similarity values computed from Eq. (2). Actually, we usually need to tune distance equations for different similarity computation approaches and even for similarity values in different ranges. For example, Edgar [11] used percent identity *D* (0≤*D*≤1) between two sequences as a similarity measure, but proposed to use two different distance computations depending on the value of *D*. In this case, Edgar computed the distance as − ln(1−*D*−*D* ^2^/5) if *D*>0.25, and retrieved the distance value from a pre-computed lookup table, otherwise. Hence, it may be beneficial to design some new distance computation equations that better match our approach. Finally, considering the generality and fast speed of our heuristic, we would expect that related research in bioinformatics and computational biology could benefit from our algorithm.
